# Labor dystocia and risk of histological chorioamnionitis and funisitis: a study from a single tertiary referral center

**DOI:** 10.1186/s12884-021-03719-3

**Published:** 2021-03-30

**Authors:** Hyo Kyozuka, Tuyoshi Murata, Toma Fukuda, Erina Suzuki, Riho Yazawa, Shun Yasuda, Aya Kanno, Akiko Yamaguchi, Yuko Hashimoto, Keiya Fujimori

**Affiliations:** 1 Department of Obstetrics and Gynecology, Fukushima Medical University School of Medicine, 1 Hikarigaoka, Fukushima, 960-1295 Japan; 2 Department of Diagnostic Pathology, Fukushima Medical University School of Medicine, 1 Hikarigaoka, Fukushima, 960-1295 Japan

**Keywords:** Labor dystocia, Intrauterine inflammation, Histological chorioamnionitis, Funisitis, Staging

## Abstract

**Background:**

Intrauterine inflammation affects short- and long-term neonatal outcomes. Histological chorioamnionitis and funisitis are acute inflammatory responses in the fetal membranes and umbilical cord, respectively. Although labor dystocia includes a potential risk of intrauterine inflammation, the risk of histological chorioamnionitis and funisitis of labor dystocia has not been evaluated yet. This study aimed to examine the association between labor dystocia and risk of histological chorioamnionitis and funisitis.

**Methods:**

In this retrospective cohort study, the cases who underwent histopathological examinations of the placenta and umbilical cord at Fukushima Medical University Hospital, Japan, between 2015 and 2020, were included. From the dataset, the pathological findings of the patients with labor dystocia and spontaneous preterm birth were reviewed. Based on the location of leukocytes, the inflammation in the placenta (histological chorioamnionitis) and umbilical cord (funisitis) was staged as 0–3. Multiple logistic regression analysis was performed to evaluate the risk of histological chorioamnionitis, histological chorioamnionitis stage ≥2, funisitis, and funisitis stage ≥2.

**Result:**

Of 317 women who met the study criteria, 83 and 144 women had labor dystocia and spontaneous preterm birth, respectively, and 90 women were included as controls. Labor dystocia was a risk factor for histological chorioamnionitis (adjusted odds ratio, 6.3; 95% confidential interval, 1.9–20.5), histological chorioamnionitis stage ≥2 (adjusted odds ratio, 6.0; 95% confidence interval, 1.7–21.8), funisitis (adjusted odds ratio, 15.4; 95% confidence interval, 2.3–101.3), and funisitis stage ≥2 (adjusted odds ratio, 18.5; 95% confidence interval, 2.5–134.0). Spontaneous preterm birth was also a risk factor for histological chorioamnionitis (adjusted odds ratio, 3.7; 95% confidence interval, 1.7–7.8), histological chorioamnionitis stage ≥2 (adjusted odds ratio, 3.0; 95% confidence interval, 1.2–7.9), and funisitis (adjusted odds ratio, 6.6; 95% confidence interval, 1.4–30.6). However, the adjusted odds ratio was smaller in spontaneous preterm births than in labor dystocia.

**Conclusion:**

Labor dystocia is a risk factor for severe histological chorioamnionitis and funisitis. Further studies are required to evaluate the effects of histological chorioamnionitis and funisitis on long-term neonatal outcomes.

## Background

Chorioamnionitis (CAM) is strongly related to preterm birth (PTB), which can lead to significant neonatal morbidity and mortality, including periventricular leukomalacia, bronchopulmonary dysplasia, pneumonia, and cerebral palsy [1–3]. Histological CAM (hCAM) is the maternal response to inflammatory stimuli in the amniotic cavity [4, 5] and is diagnosed pathologically after delivery. hCAM is not always associated with fetal infection or fetal inflammatory response syndrome. Funisitis, an inflammatory process involving the umbilical cord, is an accepted hallmark of fetal inflammatory response syndrome. Although both hCAM and funisitis are acute inflammatory lesions with important short- and long-term clinical significance [6], funisitis is associated with higher rates of neonatal morbidity and multi-organ fetal involvement than CAM [7, 8].

Recently, the descriptive term “intrauterine inflammation or infection or both” abbreviated as “Triple I” has been proposed by the National Institute of Child Health and Human Development expert panel to replace the term CAM [9]. This new term indicates that hCAM and funisitis do not always occur along with PTB and CAM is sometimes diagnosed based on the presence of inflammation of the placenta or umbilical cord without clinical symptom of infection.

Our previous study that examined the association between the fetal heart rate and risk of intrauterine inflammation in cases of PTB showed that vaginal delivery increased the risk of hCAM [10]. Therefore, uterine contractions (UC) might be a risk factor for intrauterine inflammation. Labor dystocia (LD) is the arrest of labor and one of the commonest obstetric complications in primiparous women that justifies medical intervention during labor [11, 12]. LD increases the risk of operative vaginal delivery, cesarean section (CS), and postpartum hemorrhage [13, 14]. While UCs could increase the risk of intrauterine inflammation, the effects of LD on hCAM and funisitis are not well known. Hence, the present study aimed to examine whether LD is a risk factor for hCAM and funisitis. Specifically, we have focused on the following two questions in this study: (i) does the risk of hCAM and funisitis differ between LD and spontaneous PTB (SPTB) cases and (ii) does LD affect the severity of inflammation in the placenta or the umbilical cord?

## Methods

### Study patients

In this retrospective cohort study, data from women whose placenta and umbilical cord were histopathologically examined at Fukushima Medical University Hospital, Fukushima, Japan, between July 1, 2015, and June 30, 2020, were assessed. Information on maternal and obstetric outcomes was retrieved from the medical records. In our institution, histopathological examinations of the placenta and umbilical cord are usually conducted in the following conditions: 1) SPTB before 37 weeks gestation; 2) hypertensive disorders of pregnancy; 3) clinical CAM in delivery after 37 weeks gestation; 4) placental abruption; 5) placenta accreta spectrum; 6) abnormal fetal heart rate pattern; 7) fetal abnormality, such as fetal growth restriction and fetal anomaly, which includes medically induced PTB; 8) intrauterine fetal death; and 9) LD. SPTB is defined as delivery before 37 weeks gestation due to UC or premature rupture of membranes. Hypertensive disorders of pregnancy is defined as new onset of hypertension (blood pressure ≥ 140/90 mmHg) after conception. Clinical CAM is diagnosed according to the Lencki criteria (maternal fever with positive findings on blood investigations, presence of discharge, abdominal pain, or maternal tachycardia) [15]. Abnormal fetal heart rate pattern is defined as Category III, which is proposed by the National Institute of Child Health and Human Development workshop [16]. LD is defined as follows: a case with cephalic presentation without a history of CS and (1) operative vaginal delivery with several trials of maternal effort due to arrest of the active phase for at least 4 h when the dilation of the cervix was 10 cm with or without an augmentation agent or (2) no progress with cervical dilation despite clinically adequate effective labor irrespective of augmentation, such as the use of oxytocin or amniotomy. Women with multiple gestations, delivery before 22 weeks gestation, clinical CAM after 37 weeks gestation, hypertensive disorders of pregnancy, placental abruption, placenta accreta, abnormal fetal heart rate pattern, and intrauterine fetal death were excluded from the present analysis.

Information on maternal and obstetric outcomes was retrieved from the medical records. Maternal information included maternal age (categorized as < 30, 30–39, 35–39, and ≥ 40 years) and gestational age at the time of examination. Gestational age was determined at an early stage of pregnancy based on the last menstrual period and/or ultrasound examination findings. Duration of labor was defined as the presence of true labor pain until the end of their delivery.

### Histological examination of the placenta and umbilical cord

In our institution, histological examinations of the placenta and umbilical cord were routinely conducted for the cases mentioned above. The following sites were sampled: the chorion-amnion, chorionic plate, and umbilical cord. In our institution, we usually sample from about 10 points; 2 cross sections of the umbilical cord, one from near the cord insertion and another approximately 5 cm from the umbilical cord Insertion to placenta. A one roll of the extraplacental membranes, 5 to 7 other placental sample each containing a full thickness section near by placenta to cord insertion, and other significant lesions such as hemorrhage and ischemia. These samples were fixed in 10% neutral buffered formalin and embedded in paraffin. Sections of tissue blocks were stained with hematoxylin and eosin. Based on the location of leukocytes, inflammation in the placenta and umbilical cord was staged according to Amsterdam Placental Workshop Group Consensus Statement (Table 1) [17].

**Table 1:** Inflammatory staging of the umbilical cord and placenta [17]

Score of the umbilical cord	
Stage	Interpretation
1	Chorionic vasculitis or umbilical phlebitis
2	Involvement of the umbilical vein and one or more umbilical arteries
3	Necrotizing funisitis
**Score of the placenta**	
**Stage**	**Interpretation**
1	Acute subchorionitis or chorionitis
2	Acute chorioamnionitis: polymorphonuclear leukocytes extend into fibrous chorion and/or amnion
3	Necrotizing chorioamnionitis: karyorrhexis of polymorphonuclear leukocytes, amniocyte necrosis, and/or amnion basement membrane hypereosinophilia

### Statistical analyses

In the present study, cases of fetal abnormality were used as controls. Since SPTB is known to be associated with intrauterine inflammation, we assessed the risk of intrauterine inflammation in cases with LD in comparison to the risk of not only the controls but also cases with SPTB. Due to the present study’s retrospective design, as many participants as possible who were examined during this study period were included.

Maternal information and obstetric outcomes of the LD, SPTB, and control groups were summarized. The labor duration was compared among cases classified based on the presence of intrauterine inflammation (hCAM, hCAM stage≥2, funisitis, and funisitis stage≥2). Finally, we determined the risk hCAM and funisits due to LD and SPTB by performing a logistic regression analysis, accounting for maternal age (< 30 years as reference), method of delivery (vaginal delivery or CS), gestational age (continuous variable), duration of labor (continuous variable), and parity (multipara or primiparous). SPSS v26 (IBM Corp., Armonk, NY, USA) was used for the statistical analyses. Mann–Whitney U tests and one-way analysis of variance were used to compare the continuous variables. Chi-squared tests were used to compare the categorical variables. To calculate the adjusted odds ratios (aORs) and 95% confidence intervals (CIs), a multiple logistic regression model was used. The level of statistical significance was set at *P* < 0.05.

## Results

**Fig. 1:**
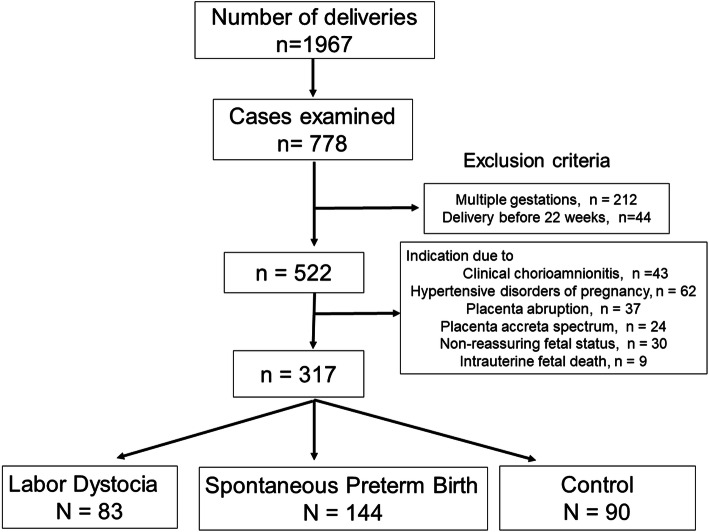
A flow chart depicting the study design

Fig. 1 shows the inclusion of the study participants. During the study period, there were 1967 deliveries at Fukushima Medical University Hospital. Among them, histopathological examination of the placenta was performed in 778 cases; of these, 212 and 44 cases were excluded because of multiple pregnancies and delivery before 22 weeks, respectively. Furthermore, 43, 62, 37, 24, 30, and 9 cases were excluded because of clinical CAM, hypertensive disorders of pregnancy, placental abruption, placenta accreta spectrum, non-reassuring fetal status, and intrauterine fetal death, respectively. As a result, 317 cases were eligible for the final analysis. They were categorized as “LD” (*n* = 83), “SPTB” (*n* = 144), and controls (*n* = 90), which included 53 and 37 cases of fetal anomaly and growth restriction and delivery without UC, respectively.

Table 2 shows the basic characteristics of the women in the three groups. Both mean maternal age and maternal age category were significantly different among the groups. The LD group tended to have more patients aged 30–39 years. The rates of primiparous and CS delivery in the LD group were 91.6 and 83.1%, respectively, and were significantly higher than those of the SPTB and control groups (*P* < 0.01 and *P* < 0.05, respectively). The mean (standard deviation) gestational age at delivery in patients in the LD, SPTB, and control groups was 39.6 (1.2), 30.7 (5.1), and 35.9 (3.4) weeks, respectively, which were significantly different (*P* < 0.001). Although the incidence of umbilical cord artery (UmA) pH < 7.20 was not significantly different among the three groups (*P* = 0.310), the mean pCO_2_, pO_2_, and base excess (BE) in the UmA were significantly different *(P* < 0.05, *P* < 0.01, and *P* < 0.01, respectively).

**Table 2:** Basic maternal characteristics of the LD, SPTB, and control groups

	Participants			
	LD	SPTB	Control	
Variable	*n* = 83	*n* = 144	*n* = 90	*P*-value
Maternal age, years	34.2 (5.8)	31.9 (6.2)	31.4 (6.1)	< 0.01^a^
Maternal age category, %				
< 30 years	24.1	34.7	38.9	< 0.05^b^
30–39 years	54.2	56.9	52.2	
> 40 years	21.7	8.3	8.9	
Maternal height, cm	157 (6.0)	158 (5.9)	158 (5.8)	0.343^a^
Primiparous, %	91.6	47.2	58.9	< 0.01^b^
Cesarean section, %	83.1	64.6	66.7	< 0.05^b^
Gestational age at delivery, weeks	39.6 (1.2)	30.7 (5.1)	35.9 (3.4)	< 0.01^a^
Birth weight at delivery, g	3143 (398)	1637 (836)	2160 (770)	< 0.01^a^

Results are expressed as mean (standard deviation) unless specified otherwise

*LD* labor dystocia, *SPTB* spontaneous preterm birth

^a^*P*-value: one-way analysis of variance

^b^*P*-value: chi-squared test

Table 3 shows the incidence of hCAM and funisitis and their staging in the LD, SPTB, and control groups. The incidence of hCAM and funisitis in the control group was 14.4 and 3.4%, respectively. The incidence of hCAM and funisitis in the LD group was 63.9 and 39.2%, respectively, which were significantly higher than those of the other two groups. The incidence of hCAM and funisitis ≥stage 2 were also the highest in the LD group (54.2 and 32.1%, respectively).

**Table 3:** The stage of CAM and funisitis in LD, SPTB, and control groups

Variable	Participants			*P*-value^a^
	LD	SPTB	Control	
	*n* = 83	*n* = 144	*n* = 90	
CAM, %	63.9	52.1	14.4	< 0.01
CAM stage, %				
Not presence	36.1	47.9	85.6	< 0.01
1	9.6	14.6	6.7	
2	24.1	13.2	1.1	
3	30.1	24.3	6.7	
CAM stage ≥2, %	54.2	37.5	7.8	< 0.01
Funisitis, %	39.2	24.8	3.4	< 0.01
Funisitis stage, %				
Not presence	61.7	75.2	97.7	< 0.01
1	4.9	3.5	0.0	
2	32.1	18.4	2.3	
3	1.2	2.8	0.0	
Funisitis stage ≥2, %	32.1	21.3	2.3	< 0.01

*CAM* chorioamnionitis, *LD* labor dystocia, *SPTB* spontaneous preterm birth

^a^*P*-value: chi-squared test

Table 4 shows the comparisons of the results of the UmA gas analysis at delivery between the LD and control groups. Both the mean UmA pH and incidence of UmA pH < 7.20 were not significantly different between the two groups (*P* = 0.465 and *P* = 0.213, respectively). The mean UmA CO_2_ was significantly higher in the control group (44.7 vs. 48.6 mmHg, respectively, *P* < 0.01), whereas the mean BE was significantly higher in the LD group (− 5.1 vs. -4.0, respectively, *P* < 0.01).

**Table 4:** Comparison of UmA gas analysis at delivery between LD and control groups

Variable	Participants		*P*-value
	LD	Control	
	*n* = 83	*n* = 87	
pH	7.30 (0.06)	7.29 (0.07)	0.465^a^
pH < 7.20, %	3.6	9.2	0.213^b^
pCO_2_, mmHg	44.7 (8.0)	48.6 (10.0)	< 0.01^a^
pO_2_, mmHg	17.7 (6.0)	17.4 (6.6)	0.781^a^
BE, mmol/L	−5.1 (2.8)	−4.0 (3.2)	< 0.05^a^

Results are specified as mean (standard deviation) unless specified otherwise

LD: labor dystocia, BE: base excess, UmA: Umbilical artery

^a^*P*-value: t-test

^b^*P*-value: chi-squared test

Table 5 shows the comparisons of labor duration between LD cases classified based on the presence or absence of histological inflammation. Mann–Whitney U test showed that there was no significant difference in the labor duration between the cases with and without inflammation, such as hCAM, hCAM stage≥2, funisitis, and funisitis stage≥2 (*P* = 0.837, *P* = 0.790, *P* = 0.476, and *P* = 0.211, respectively).

**Table 5:** Comparison of labor duration based on the presence or absence of intrauterine inflammation in LD

	Positive	Negative	*P*-value^a^
CAM, n	51	30	
hours, median (IQR)	9 (6–14)	8 (7–14)	0.837
CAM stage≥2, n	44	37	
hours, median (IQR)	9 (6–14)	8 (7–15)	0.79
Funisitis, n	33	48	
hours, median (IQR)	9 (6–14)	9 (7–14)	0.476
Funisitis stage≥2, n	29	52	
hours, median (IQR)	9 (6–14)	9 (7–17)	0.211

*LD* labor dystocia, *CAM* chorioamnionitis, *IQR* interquartile range

^a^*P*-value: Mann–Whitney U test

Table 6 shows the association between LD, SPTB, and intrauterine inflammation. LD was a risk factor for hCAM (aOR: 6.3, 95% CI: 1.9–20.5), hCAM stage≥2 (aOR: 6.0, 95% CI: 1.7–21.8), funisitis (aOR: 15.4, 95% CI: 2.3–101.3), and funisitis stage≥2 (aOR: 18.5, 95% CI: 2.5–134.0). SPTB was also a risk factor for hCAM (aOR: 3.7, 95% CI: 1.7–7.8), hCAM stage≥2 (aOR: 2.8, 95% CI: 1.2–7.9), and funisitis (aOR: 6.6, 95% CI: 1.4–30.6).

**Table 6:** The risk of hCAM and funistis in LD and SPTB

	hCAM	hCAM ≥2	Funisitis	Funisitis ≥2
LD (+) OR (95% CI)	10.5 (5.0–21.9)	14.1 (5.8–34.0)	26.3 (6.1–114.8)	20.1 (4.6–88.1)
LD (+) aOR (95% CI)	6.3 (1.9–20.5)	6.0 (1.7–21.8)	15.4 (2.3–101.3)	18.5 (2.5–134.0)
SPTB (+) OR (95% CI)	6.0 (3.1–11.5)	6.3 (2.8–13.9)	14.0 (3.3–60.0)	11.5 (2.7–49.4)
SPTB (+) aOR (95% CI)	3.7 (1.7–7.8)	3.0 (1.2–7.9)	6.6 (1.4–30.6)	3.6 (0.7–17.6)

*hCAM* histological chorioamnionitis, *LD* labor dystocia, *SPTB* spontaneous preterm birth, *OR* odds ratio, *aOR* adjusted odds ratio, *CI* confidential interval

aOR was calculated accounting for maternal age (< 30 years as reference), parity (0: primiparous, 1: multipara), mode of delivery (0: cesarean delivery, 1: vaginal delivery), gestational age (continuous variable), and duration of labor (continuous variable)

## Discussion

This is the first study to examine the correlation between LD and risk of intrauterine inflammation in both hCAM and funisitis cases. Since we did not have histopathological information on cases with so-called normal progress of labor, we defined those with fetal abnormalities as controls and also examined the association between SPTB and intrauterine inflammation to compare the degree of risk of intrauterine inflammation between the LD and SPTB cases. We found a significant difference in maternal age, parity, mode of delivery, and gestational age at delivery among the three groups. After accounting for confounding factors, we found that SPTB was associated with hCAM, hCAM stage≥2, and funisitis. We also found the association of LD with hCAM, hCAM stage≥2, funisitis, and funisitis stage≥2. The magnitude of aOR in LD and SPTB for each intrauterine inflammation event (6.3 and 3.7 for hCAM, 6.0 and 3.0 for hCAM stage≥2, and 15.4 and 6.6 for funisitis, respectively) indicated that the risk of inflammation was higher in LD than in SPTB.

Our finding that LD, which is characterized by ineffective UC, is associated with hCAM and funisitis is in accordance with that of a previous study. Lee et al. reported that the frequencies of hCAM and funisitis in spontaneous term deliveries were 23.6% (310/1316) and 6.7% (88/1316), respectively. They also reported the association of longer labor duration, primiparous, rupture of membranes, and higher gestational age with both hCAM and funisitis in term spontaneous deliveries [18]. However, reports on the association between UC and funisitis are conflicting. Seong et al. reported that labor was associated with an increased risk of microbial invasion of the amniotic cavity and hCAM in term pregnancies. They also reported that the more advanced the cervical dilatation, the greater is the risk of microbial invasion of the amniotic cavity and hCAM. However, the risk of funisitis was not reported to increase with the presence of labor or as a function of cervical dilatation [19].

Compared to the previous study, we used the staging system based on Amsterdam Placental Workshop Group Consensus Statement [17]. Consequently, we found that LD was more strongly associated with hCAM and funisitis compared with SPTB by mean of magnitude of aOR. However, we did not assessed the severity of inflammation which is defined using a grading system in the Amsterdam Placental Workshop Group Consensus Statement [17], further studies are required to validate if pathological staging system of inflammation based on the Amsterdam Placental Workshop Group Consensus Statement [17] in LD is a reliable method to provide clinical meaning to neonatal outcomes.

Our findings revealed higher pCO_2_ in controls that could lead to respiratory acidosis and lower BE in LD cases that could lead to metabolic acidosis. These results are biologically plausible because our previous animal study with an intrauterine inflammation pregnant sheep model proved that the histological inflammation directly caused metabolic acidosis, and not respiratory acidosis [20].

Previously, we have reported that labor UC and immature gestational age at delivery were related to hCAM in SPTB cases [10]. The reason why UC leads to intrauterine inflammation is still unclear. One reason may be that the vaginal fluid is drawn into the uterine cavity by UCs, thus resulting in intrauterine inflammation. A previous study (involving sonohysterography with contrast media) reported that UC acted as a peristaltic pump by which the vaginal fluid could ascend into the uterine cavity [21]. Therefore, exposure to non-effective UC could be a potential risk factor for intrauterine inflammation. Kim et al. reported that intra-amniotic inflammation was present in 12% of patients with regular UC without cervical changes, while culture-proven intra-amniotic infection was present in 3% of the patients [22]. Therefore, intrauterine inflammation due to UC should be interpreted cautiously because inflammation could occur without an intrauterine infection, which is typical of intrauterine inflammation [23].

Recently, the mean maternal age and ratio of nulliparous cases are increasing in Japan [24]. These cases are more likely to present with LD [14, 25, 26] and underline the necessity of obstetrical management not only because LD increases maternal morbidity, such as postpartum transfusion, third- or fourth-degree perineal lacerations, and emergency cesarean or operative vaginal delivery [27], but also because intrauterine inflammation could affect the offspring’s health in the long term. Therefore, obstetricians need to pay increased attention to cases with a potential risk for LD to prevent intrauterine inflammation.

The main strength of the present study is that the data were derived from a single tertiary care fetal medicine unit where all women who delivered were managed using approximately the same protocol. The other strength is the information regarding the severity of inflammation using the Blanc/Nakayama classification. Evidence suggests that an enhanced fetal cytokine release as part of the fetal inflammatory response syndrome may directly injure the developing brain [28]; therefore, we will examine the relationship between the degree of inflammation due to LD and long-term neonatal outcomes in future studies.

The present study has some limitations. First, we do not have information on the maternal or neonatal blood tests performed at the time of delivery, because we do not routinely perform such tests except in cases of suspected clinical CAM. Therefore, we categorized the cases with maternal fever as clinical CAM and excluded them from the present analysis. Second, we chose cases with fetal abnormality as the controls because we do not routinely perform placental histopathological examination in healthy cases. Therefore, they may not represent the truly healthy cases. For example, the mean gestational age (35.9 weeks) and the ratio of CS (66.7%) in the control group were quite different compared with the Japanese epidemiological data during the same period at the same place [29]. To minimize this limitation, we also calculated the risk of intrauterine inflammation in the SPTB group and compared the aORs between the cases with LD and those with SPTB. Third, although we accounted for the duration of labor in the logistic model, we did not classify LD into dystocia during the latent and active phases. Fourth, we did not conduct a power analysis for the appropriate number of patients required in this study, and it is unclear whether the sample size was sufficient to achieve statistical significance. Finally, although we excluded cases with maternal fever, we did not have information on the culture tests of the vaginal or amniotic fluids, which could determine whether the pathological inflammation was due to either infection or non-specific inflammation [30].

## Conclusion

Compared with SPTB, LD is a potential risk factor for intrauterine inflammation, especially in severe cases; therefore, obstetricians must pay attention to pregnant women at a potential high risk for LD. Further studies that examine the long-term neonatal outcomes in patients with intrauterine inflammation due to LD are required.

## Data Availability

The data that support the findings of this study are available from the corresponding author upon reasonable request.

## Abbreviations

CAM: Chorioamnionitis
PTB: Preterm birth
hCAM: Histological CAM
LD: Labor dystocia
aOR: Adjusted odds ratio
CI: Confidential interval
SPTB: Spontaneous preterm birth
CS: Cesarean section
UC: Uterine contraction
